# FAT4 silencing promotes epithelial-to-mesenchymal transition and invasion via regulation of YAP and β-catenin activity in ovarian cancer

**DOI:** 10.1186/s12885-020-06900-7

**Published:** 2020-05-04

**Authors:** Shika Hanif Malgundkar, Ikram Burney, Mansour Al Moundhri, Moza Al Kalbani, Ritu Lakhtakia, Aikou Okamoto, Yahya Tamimi

**Affiliations:** 1 Departments of Biochemistry, Obstetrics & Gynecology, College of Medicine and Health Sciences, Sultan Qaboos University, PO Box 35, PC 123 Muscat, Sultanate of Oman; 2 Departments ofMedicine, and Obstetrics & Gynecology, College of Medicine and Health Sciences, Sultan Qaboos University, PO Box 35, PC 123 Muscat, Sultanate of Oman; 3 Obstetrics & Gynecology, College of Medicine and Health Sciences, Sultan Qaboos University, PO Box 35, PC 123 Muscat, Sultanate of Oman; 4 Department of Pathology, College of Medicine, Mohammed Bin Rashid University of Medicine and Health Sciences, Dubai, UAE; 5 Department of Obstetrics and Gynecology, The Jikei University School of Medicine, Tokyo, Japan

**Keywords:** *FAT4*, Silencing, Invasion, *YAP*, Ovarian cancer

## Abstract

**Background:**

The adhesion molecule, FAT4, has a tumor suppressor function with a critical role in the epithelial-to-mesenchymal-transition (EMT) and anti-malignant growth in several cancers. No study has investigated yet its role in epithelial ovarian cancer (EOC) progression. In the present study, we examined the role of *FAT4* in proliferation and metastasis, and its mechanisms of interaction in these processes.

**Methods:**

We have performed cell viability, colony formation, and invasion assays in ovarian cancer cells treated with siRNA to knockdown *FAT4* gene expression. The regulatory effects of FAT4 on proteins involved in apoptotic, Wnt, Hippo, and retinoblastoma signaling pathways were evaluated by Western blotting following FAT4 repression. Also, 426 ovarian tumor samples and 88 non-tumor samples from the Gene Expression Profiling Interactive Analysis (GEPIA) database were analyzed for the expression of *FAT4*. Pearson’s correlation was performed to determine the correlation between *FAT4* and the *E2F5*, *cyclin D1*, *cdk4*, and *caspase 9* expressions.

**Results:**

Lower expression of FAT4 was observed in ovarian cancer cell lines and human samples as compared to non-malignant tissues. This down-regulation seems to enhance cell viability, invasion, and colony formation. Silencing *FAT4* resulted in the upregulation of *E2F5*, vimentin, YAP, β-catenin, cyclin D1, cdk4, and Bcl2, and in the downregulation of GSK-3-β, and caspase 9 when compared to control. Furthermore, regulatory effects of FAT4 on the EMT and aggressive phenotype seem to occur through Hippo, Wnt, and cell cycle pathways.

**Conclusion:**

*FAT4* downregulation promotes increased growth and invasion through the activation of Hippo and Wnt-β-catenin pathways.

## Background

Epithelial Ovarian Cancer (EOC) is a malignant disease originating from the outer surface cells covering the ovary [1]. It represents 90% of all types of ovarian cancer [2] and is considered to be the 8th leading cause of cancer-related deaths among women worldwide [3]. Due to its asymptomatic nature and the absence of reliable biological markers for early detection [4], only 20% of ovarian tumors are diagnosed at early stages I/II, while the majority are diagnosed at advanced stages (III to IV) [5]. An increase in the incidence rate, and also the shift towards a younger affected population was recently noticed [3].

Despite the broad advances in gynecological research, the cascade of events leading to epithelial ovarian cancer remains ambiguous, and efforts are needed to elucidate the underlying mechanisms. To identify candidate genes with a potential role in the pathogenesis of ovarian cancer, we performed chromatin immunoprecipitation (ChIP) using *E2F5,* a transcription factor highly expressed in early stages of EOC [6]. ChIP data revealed that *FAT4* was one of the immunoprecipitated downstream genes regulated by *E2F5*, and thus can be suspected to have a role in EOC pathogenesis.

FAT tumor suppressor homolog 4 (FAT4), is a member of the FAT family that consists of (FAT1–4) and encodes a single transmembrane protein containing 32–34 extracellular cadherin repeats, a transmembrane domain and a cytoplasmic domain [7]. *FAT4* was identified as a tumor suppressor in mouse mammary epithelial cell line and triple-negative breast cancer [8–11].

There is increasing evidence of a possible relation between the *FAT4* downregulation and the pathogenesis of several malignancies, including breast, colorectal, and gastric cancers [8, 12, 13]. Also, previous mutational screening studies revealed missense and nonsense mutations of *FAT4* in hepatocellular (10%) [14], pancreatic (8%) [15], head-and-neck squamous cell cancers (6%) [16], endometrioid, and mucinous primary ovarian tumors (15%) [17].

In endometrial cancer, *FAT4* downregulation was attributed to the silencing of USP51, a de-ubiquitinating enzyme, suggested as a direct interacting partner of *FAT4*, contributing to its tumor suppressor role [18]. While in colorectal cancer, *FAT4* was found to inhibit tumorigenesis by regulating the PI3K activity in the PI3K/AKT/mTOR signaling pathway and to play a significant role in preventing the epithelial-to-mesenchymal transition (EMT) [13]. The EMT is a crucial step for several developmental processes and a genuine hallmark for aggressive phenotype and invasion [19, 20]. Moreover, in gastric cancer, *FAT4* silencing stimulated cell proliferation, migration, and cell cycle progression through the nuclear translocation of YAP [21]. Hence, *FAT4* was found to regulate the downstream effectors of the Hippo pathway, YAP/TAZ [18, 21, 22]. Alternatively, YAP activity is regulated by the core Hippo kinases. Phosphorylation of YAP results in its cytoplasmic retention and inactivation, while un-phosphorylated YAP is in its active mode, and are freely translocated into the nucleus to promote transcription of cell proliferation and anti-apoptotic genes [23]. In ovarian cancer, activated YAP was associated with poor survival by promoting cell proliferation, EMT, anchorage-independent growth, and resistance to cisplatin-induced apoptosis [24].

In the present study, we examined the role of *FAT4* downregulation in the tumorigenesis of EOC cells and its consequent impact on the expression of key proteins involved in Hippo, Wnt-β-catenin, apoptotic, EMT, and cell cycle pathways. The obtained data shed some light on the role of the FAT4 adhesion molecules in ovarian cancer tumorigenesis through different pathways, namely, Hippo, and Wnt-β-catenin.

## Methods

### Cell culture

The human ovarian cancer cell lines: MCAS and OVSAHO (JCRB cell bank, Osaka, Japan, catalog no. JCRB0240 and no. JCRB1046 respectively) were kindly provided by Prof. Aikou Okamoto (Jikei University School of Medicine, Japan), in 2016. The cisplatin sensitive A2780 (The European Collection of Authenticated Cell, ECACC catalog no. 93112519) and cisplatin-resistant A2780-cis (ECACC catalog no. 93112517) cell lines were a generous gift from Dr. Benjamin Tsang (University of Ottawa, Canada), in 2018. The transformed normal epithelial ovarian cell line HOSE6–3 (RRID: CVCL_7673), established by Prof. GSW Tsao (School of Biomedical Sciences, The University of Hong Kong), was kindly provided by his laboratory in 2018. To avoid contaminations, our cell-culture laboratories, including hoods and incubators, are systemically fumigated every year, and any new cells are tested upon arrival, for the presence of mycoplasma using the “Mycoplasma Detection Kit” (Lonza, Catalog #: LT07–118). None of the cells used for this study were tested positive.

MCAS, A2780, A2780 cis and HOSE6–3 cells were propagated in DMEM (Gibco, NY, USA), while OVSAHO was cultured in RPMI-1640 media supplemented with 10% FBS (Gibco, NY, USA) and 1% penicillin-streptomycin antibiotic (Gibco, NY, USA) in a humidified incubator at 37 °C and 5% CO_2_.

### RNA extraction and qRT-PCR

Total RNA was extracted from the ovarian cancer cell lines using PureLink RNA mini kit (Invitrogen, CA, USA) according to the manufacturer’s protocol, and subsequently reverse transcribed to complementary DNA using high capacity reverse transcription kit (Invitrogen, CA, USA). qRT-PCR was performed using TaqMan pre-optimized probes (*FAT4*: Catalogue No. Hs01570499_m1, *E2F5*: Catalogue No.Hs00231092_m1, *GAPDH*: Catalogue No. 402869; Thermo Scientific Fisher, USA) and run on ABI 7500 Fast real-time PCR machine (Applied Biosystems, Austin, TX). All the messenger RNA data were normalized to *GAPDH* expression, and relative expression was computed by the comparative Ct method.

### Cell transfection with siRNA

1.5 × 10^5^ to 3 × 10^5^ cells (MCAS and OVSAHO) were seeded in six-well plate and incubated until they reached 70% confluency. The cells were then transfected with commercially available pre-designed anti-*FAT4* expression siRNA (Catalogue No. sc-88,877, Santa Cruz Biotechnology Inc., USA), using lipofectamine RNAiMAX reagent (Invitrogen; Thermofisher Scientific, USA) and Opti-MEM medium (Invitrogen, USA) according to the manufacturers protocol. The siRNA used consists of a pool of 3 specific probes of 19–25 nucleotides long designed and pre-optimized to act on different positions along the mRNA with no off-target effects. FAT4 siRNA (35 pmol) and 7.5 μl of lipofectamine RNAiMAX reagent were used per ml of the media per well. The cells were then incubated for 36 h at 37 °C and 5% CO_2_ atmosphere before RNA and protein extraction. Cells treated with scrambled siRNA were used as a negative control.

### In vitro **cell proliferation assay**

Cell viability was assessed using Alamar blue reagent (Invitrogen, CA, USA). MCAS and OVSAHO cells were seeded in 96 well culture plates at a density of 10^4^ cells per well, and transfected 1 day later with *FAT4* siRNA, and incubated for 24–96 h at 37 °C and 5% CO_2_. Cell proliferation was measured by adding 10 μl Alamar blue dye to the culture medium and incubating for 4 h at 37 °C. The absorbance was measured at 570 nm using Multiscan spectrum spectrophotometer (Thermo Fisher Scientific Inc., MA, USA).

### Colony-forming assay

1 × 10^5^ MCAS, and OVSAHO cells treated with *FAT4-*siRNA were seeded in a six-well plate and incubated at 37 °C in a 5% CO_2_ atmosphere for 14 days; medium was refreshed every 72 h. Cells were stained with crystal violet and observed under the microscope. For soft agar colony formation assay, 10^5^ cells per well were treated with *FAT4* siRNA, and resuspended in 0.3% soft agar in culture medium and layered onto 0.6% solidified agar in a six-well plate. After incubation for 2 weeks, colonies were stained with crystal violet and counted in three random fields at 40X magnification.

### Cell invasion assay

The invasive potential of MCAS and OVSAHO cells, following the silencing of *FAT4,* was evaluated using QCM™ Collagen cell invasion assay kit 24-well 8 μm, colorimetric (Sigma Aldrich) according to the manufacturer’s protocol. Briefly, 1.25 × 10^5^*FAT4*-siRNA transfected cells suspended in 250 μl of serum-free media was added to the upper chamber of the insert. 500 μl of the medium supplemented with 10% FBS was added to the lower chamber. After incubation for 48 h, non-invading cells on the upper surface were removed with a cotton swab and cells invading to the lower chamber were stained with crystal violet, and treated with commercially provided extraction solution (QCM™ Collagen cell invasion assay kit 24-well 8 μm, colorimetric, Sigma Aldrich). The absorbance was measured at 560 nm using Multiscan spectrophotometer (Thermofisher Scientific Inc., MA, USA).

### Western blotting

*FAT4* knocked-down MCAS cells (36 h post transfection) were subjected to proteins extraction using RIPA lysis buffer (Santa Cruz Biotechnology Inc., USA) supplemented with protease and phosphatase inhibitor (Thermofisher, USA). The lysate was centrifuged, and the supernatant was recovered and stored at -80 °C. After protein quantification, 40 μg were resolved on 8% SDS-PAGE and electro-transferred onto a nitrocellulose membrane which was incubated in a blocking reagent (5% BSA in tris buffered saline/Tween-20 buffer (TBST)) for 1 h at room temperature. Then incubated with the primary antibodies directed against the following proteins Rb (sc-50, Santa Cruz Biotechnology), actin (sc-1616, Santa Cruz Biotechnology), cdk 4 (sc-260, Santa cruz Biotechnology), E2F5 (sc-999, Santa Cruz Biotechnology, USA); caspase 9 (PA5–22252, Invitrogen, Thermofisher), β-catenin (PA5–77934, Invitrogen, Thermofisher), phospho-YAP (PA5–17481, Invitrogen, Thermofisher), phospho-β-catenin (pβ-catenin; PA5–67504, Invitrogen, Thermofisher), phospho-retinoblastoma (pRb; PA5–37715, Invitrogen, Thermofisher), E cadherin (PA5–32178, Invitrogen, Thermofisher), vimentin (PA5–27231, Invitrogen, Thermofisher, USA)*, FAT4 (ab130076, abcam),* cyclin D1 (ab134175, Abcam), GSK-3-β (#9315, Cell Signaling Technology), and phospho-GSK-3-β (pGSK-3-β; #5558, Cell Signaling Technology). All the primary antibodies were used at a dilution of 1/200. Antibody against beta-actin was used as a loading control. After washing 3X5 minutes in TBST, membranes were incubated with horseradish peroxidase-conjugated secondary antibody (dilution 1:5000) for 2 h at room temperature and revealed using ECL western blotting detection reagent (Pierce Biotechnology, Illinois, USA).

### Bioinformatics & Statistical analysis

We explored “Gene Expression Profiling Interactive Analysis” (GEPIA) (http://gepia.cancer-pku.cn/), available public database to examine the expression of genes of interest at the RNA levels. We analyzed 426 ovarian tumor samples and 88 non-tumor samples for the expression of *FAT4*. Pearson’s correlation was performed to determine the correlation between *FAT4* and the *E2F5*, *cyclin D1*, *cdk4*, and *caspase 9* expressions.

Student’s t-test was performed using SPSS software (version 23, SPSS, Inc., Chicago, Il, USA) as well as in GraphPad Prism (version 8.1.2 GraphPad Software Inc., San Diego, Ca). Statistical significance was set at *p* < 0.05 [p < 0.05(*), *p* < 0.01(**), *p* < 0.001(***) and *p* < 0.0001 (****)].

## Results

### *FAT4***expression is reduced in ovarian cancer cell lines**

**Fig. 1:**
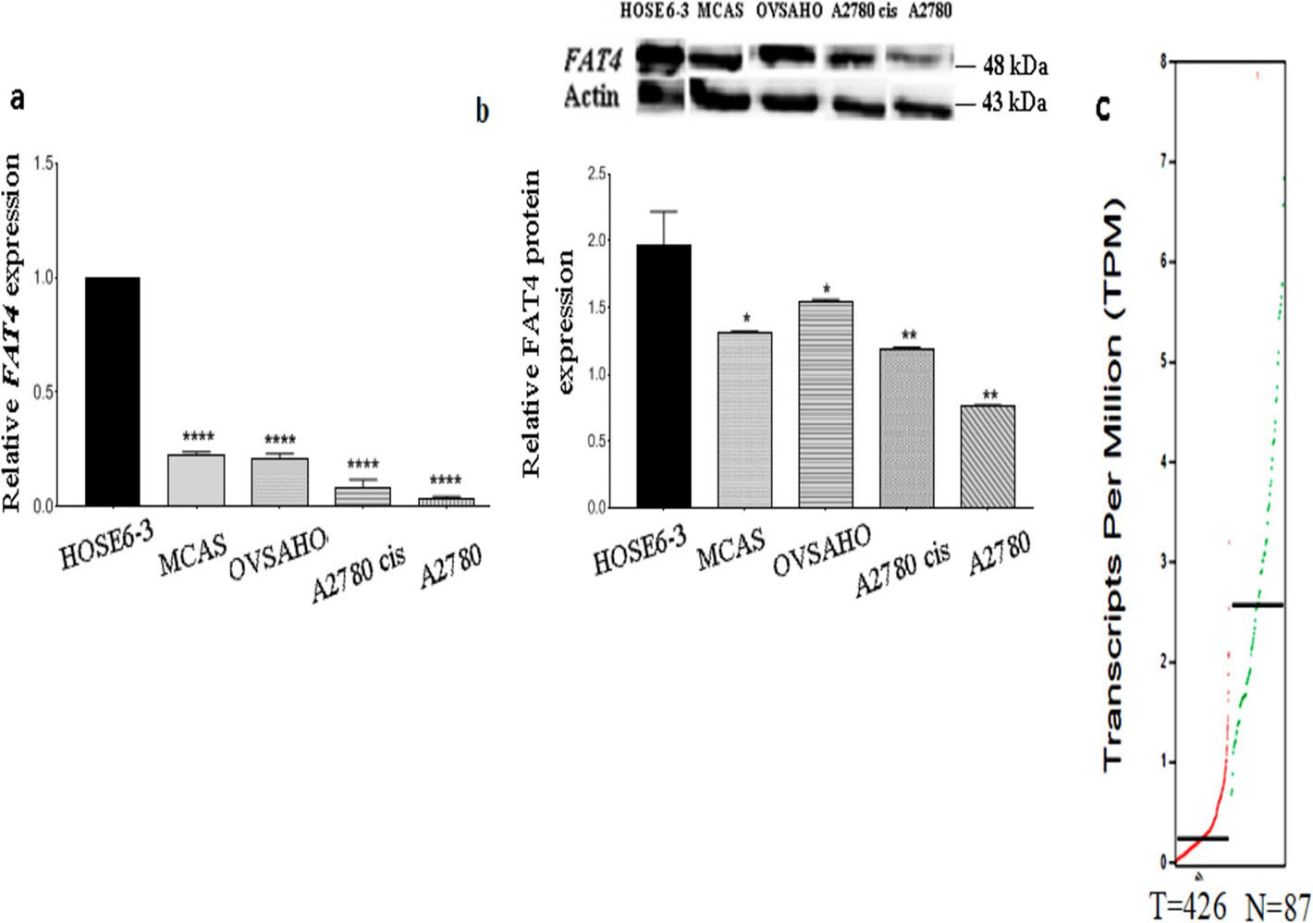
**a**. Relative expression of FAT4 at mRNA levels in ovarian cancer cell lines. MCAS and OVSAHO show high FAT4 expression as compared to A2780 and A2780 cis (*p* = 0.0001). **b**. Relative expression of FAT4 at protein levels in MCAS and OVSAHO cells. FAT4 was highly expressed in OVSAHO (*p* = 0.0459), and MCAS (*p* = 0.0107), cells while, it was least expressed in A2780 cis (*p* = 0.006) and A2780 (*p* = 0.0012) cells. Full-length blots are presented in Supplementary figure S1. **c**. Differential expression of FAT4 in tumor and healthy ovarian tissue sample from GEPIA database. FAT4 was significantly downregulated in 426 tumor samples as compared to 87 healthy samples. Red line represents tumor samples and green line represents healthy ovarian samples. Data represent mean and standard deviation from at least three independent experiments performed in triplicates

We evaluated the expression of *FAT4* in ovarian cancer cell lines (MCAS, OVSAHO, A2780, A2780 cis), and normal (HOSE 6–3) cell line, using qRT-PCR and Western Blotting. As shown in Fig. 1a, *FAT4* was significantly down-regulated at the mRNA level in A2780 and A2780 cis cell lines (*p* = 0.0001 and *p* = 0.0002 respectively). MCAS and OVSAHO displayed lower FAT4 expression (*p* = 0.0001) as compared to the normal HOSE 6–3 cell line. Similar results were obtained by western blot analysis of FAT4 protein expression in ovarian cancer cells (Fig. 1b and supplementary figure S1). FAT4 expression was the highest in HOSE 6–3 and moderately expressed in OVSAHO (*p* = 0.0459), MCAS (*p* = 0.0107), A2780 cis (*p* = 0.006) and A2780 (*p* = 0.0012) cell lines.

Analysis of *FAT4* expression in 426 ovarian tumors and 87 non-malignant samples from TCGA and GEPIA public databases revealed that *FAT4* expression was lower in ovarian tumors than in healthy tissues (Fig. 1c), which is consistent with our findings.

### *FAT4***downregulation promotes proliferation and colony formation**

**Fig. 2:**
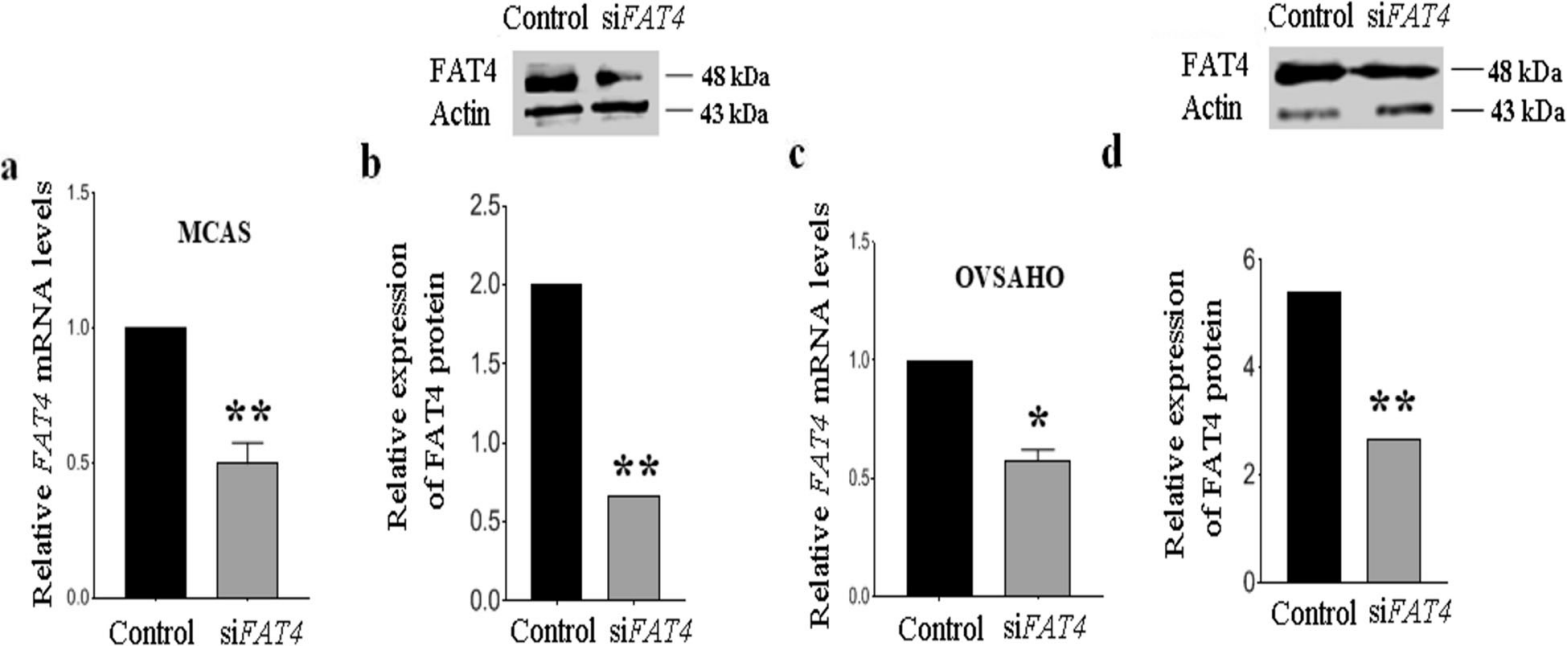
**a**. Relative expression of FAT4 in MCAS cells following its knockdown at a. mRNA level, and **b**. protein levels. FAT4 was knocked down significantly (*p* = 0.0016) in MCAS. Full-length blots are presented in Supplementary figure S1. **c**. Relative expression of FAT4 in OVSAHO cells following its knockdown at a. mRNA level, and d. protein levels. Full-length blots are presented in Supplementary figure S1. FAT4 was knocked down significantly in OVSAHO (*p* = 0.0055). Data represent mean and standard deviation from at least three independent experiments performed in triplicates

**Fig. 3:**
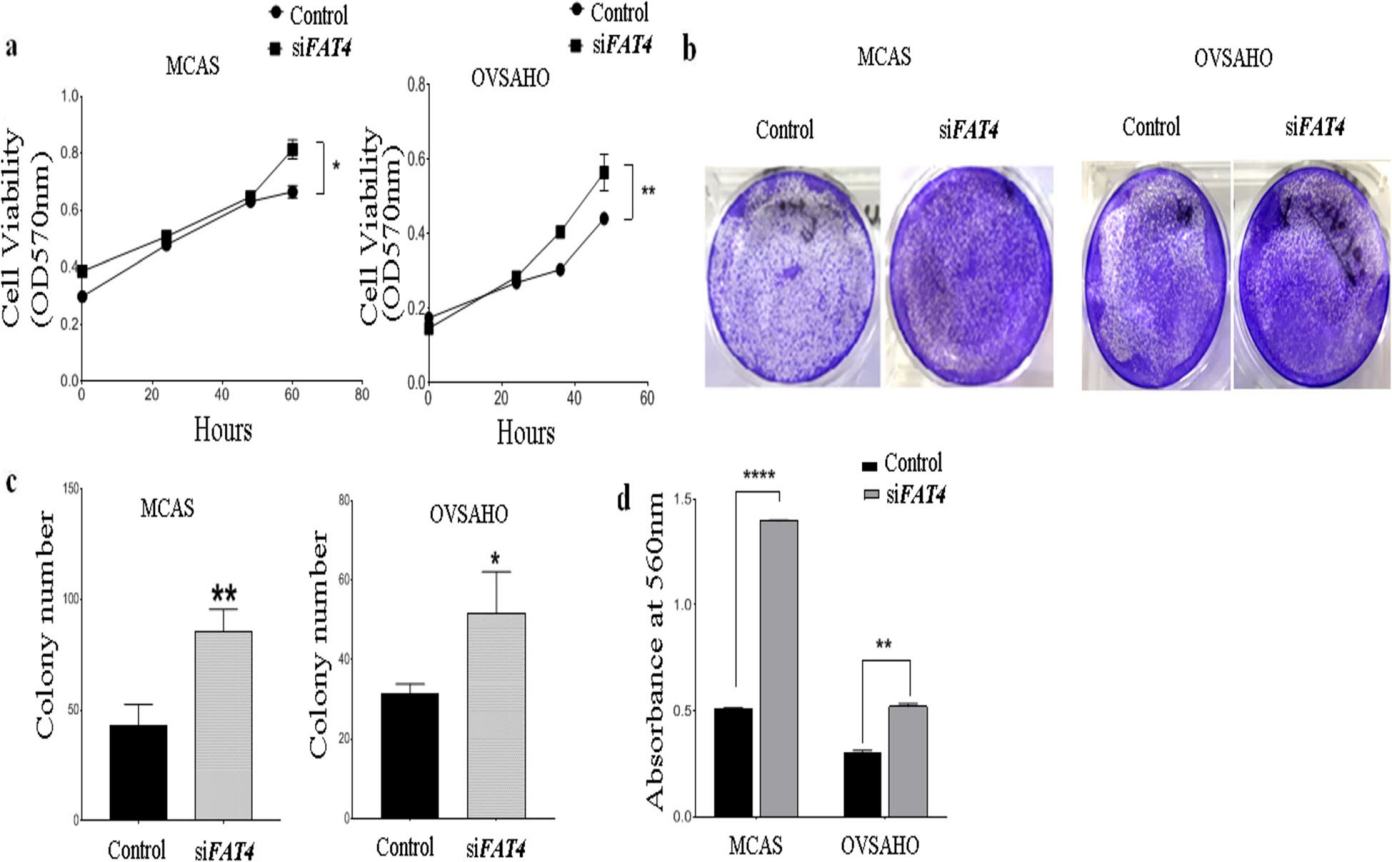
Functional role of *FAT4* on cell proliferation, colony formation, and invasive ability. **a**. Alamar blue assay displaying enhanced cell proliferation in *FAT4* knocked MCAS and OVSAHO cells (*p* = 0.0001 and *p* = 0.0054 respectively) as compared to control. **b**. Colony formation assay in control and *FAT4* siRNA treated MCAS, and OVSAHO cells. The assay shows a higher number of colonies in *FAT4* siRNA treated cells. The cells were stained with crystal violet. **c**. Soft agar colony formation assay in control and *FAT4* siRNA treated MCAS and OVSAHO cells. Anchorage-independent growth was significantly higher in cells treated with si*FAT4* MCAS cells (*p* = 0.0055) and OVSAHO cells (*p* = 0.0302). **d**. Silencing *FAT4* by siRNA promoted cell invasion in vitro in MCAS and OVSAHO cells (*p* = 0.0001 in MCAS and *p* = 0.003 in OVSAHO). MCAS and OVSAHO cells treated with scrambled siRNA were used as control. Data represent the mean and the standard deviation from at least three independent experiments performed in triplicates

To evaluate the role of *FAT4* on ovarian cancer cell proliferation, we knocked-down *FAT4* in MCAS and OVSAHO ovarian cancer cell lines, using siRNA (Figs. 2a-d and S1). The effect of silencing *FAT4* on cell growth was assessed by Alamar blue assay in MCAS and OVSAHO cells, which showed that transient inhibition of *FAT4* significantly enhanced cell proliferation (*p* = 0.0286 and *p* = 0.0054 respectively) (Fig. 3a) and colony formation ability in MCAS and OVSAHO cells compared to control (Fig. 3b and c).

### *FAT4***downregulation promotes invasion in ovarian cancer cells**

We performed cell invasion assay to investigate whether *FAT4* regulates invasiveness in ovarian cancer cell lines. Results showed that the invasive ability of MCAS and OVSAHO ovarian cancer cell lines increased significantly following the *FAT4* knockdown (*p* = 0.0001 and *p* = 0.003 respectively), suggesting its tumor-suppressive ability to prevent invasion in vitro as shown in Fig. 3d.

### *FAT4***role in the epithelial-mesenchymal transition (EMT)**

**Fig. 4:**
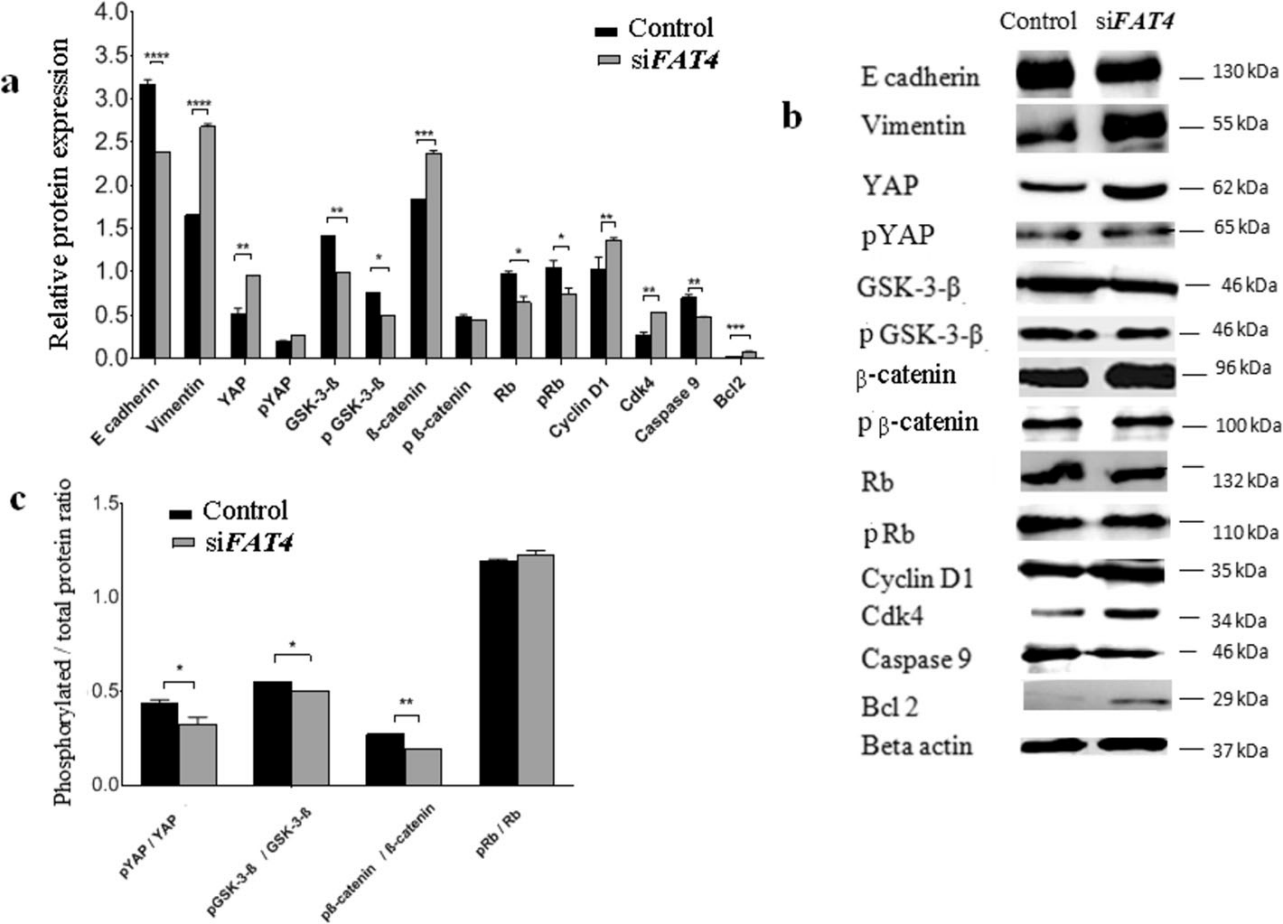
Regulatory effects of *FAT4* on the expression of proteins involved in EMT, Hippo, Wnt-β-catenin, apoptotic, and retinoblastoma pathways by Western blotting. **a**. Relative expression variation of proteins. The expression of Vimentin (*p* = 0.0001), YAP (*p* = 0.0018), β-catenin (*p* = 0.001), Bcl2 (*p* = 0.0001), cyclin D1 (*p* = 0.0017) and cdk4 (*p* = 0.0025) was higher in *FAT4* siRNA treated cells as compared to control. β-actin was used as an internal control. **b**. Western blot demonstrating bands for protein expression in control and FAT4 knocked cells. Full-length blots are presented in Supplementary figure S1. **c**. The ratio of phosphorylated to total YAP, GSK-3β, β-catenin, and retinoblastoma proteins following FAT4 repression. The pYAP/ YAP ratio was lower in *FAT4* siRNA treated cells as compared to the control (*p* = 0.0286). Similarly, pGSK-3-β/GSK-3-β ratio, and pβ-catenin/β-catenin ratio was lower in *FAT4* siRNA treated cells (*p* = 0.018, and *p* = 0.001 respectively) as compared to control. There was no significant difference in pRb/Rb ratio between *FAT4* siRNA treated cells and control. MCAS cells treated with scrambled siRNA was used as control. Data represent mean and standard deviation from at least three independent experiments performed in triplicates

We investigated the role of *FAT4* on EMT, a process which plays a crucial role in cell transformation, invasion, and metastasis by Western Blotting. Relative protein expression variation and the protein bands in control and FAT4-siRNA treated MCAS cells are shown in Figs. 4a-b and S1 respectively, while the ratio of phosphorylated to total proteins are displayed in Fig. 4c. In cells treated with *FAT4*-siRNA, we observed a reduced expression of the epithelial marker, E-cadherin. In contrast, the expression of the mesenchymal marker, vimentin was enhanced in *FAT4*-siRNA treated cells as compared to non-treated cells (*p* < 0.0001).

### *FAT4***regulatory effects on hippo, apoptotic, cell cycle, and Wnt signaling pathways**

We hypothesized that *FAT4* exerts its effect on the tumor characteristics of ovarian cancer cells through Hippo, apoptotic, retinoblastoma, and Wnt pathways. Based on this, we aimed to detect the expression of crucial proteins involved in each of these pathways through western blotting (Fig. 4), performed on MCAS cells treated with *FAT4-*siRNA. The expression of YAP (*p* = 0.0018), β-catenin (*p* = 0.001), cyclin D1 (*p* = 0.0025), cdk4 (*p* = 0.0017) and Bcl2 (*p* = 0.0001), were significantly higher in *FAT4*-siRNA treated cells as compared to the control. In contrast, the expression of GSK-3-β (*p* = 0.0013), Rb (*p* = 0.0028), pRb (*p* = 0.0491), and caspase 9 (*p* = 0.001) was lower when compared to the control.

**Fig. 5:**
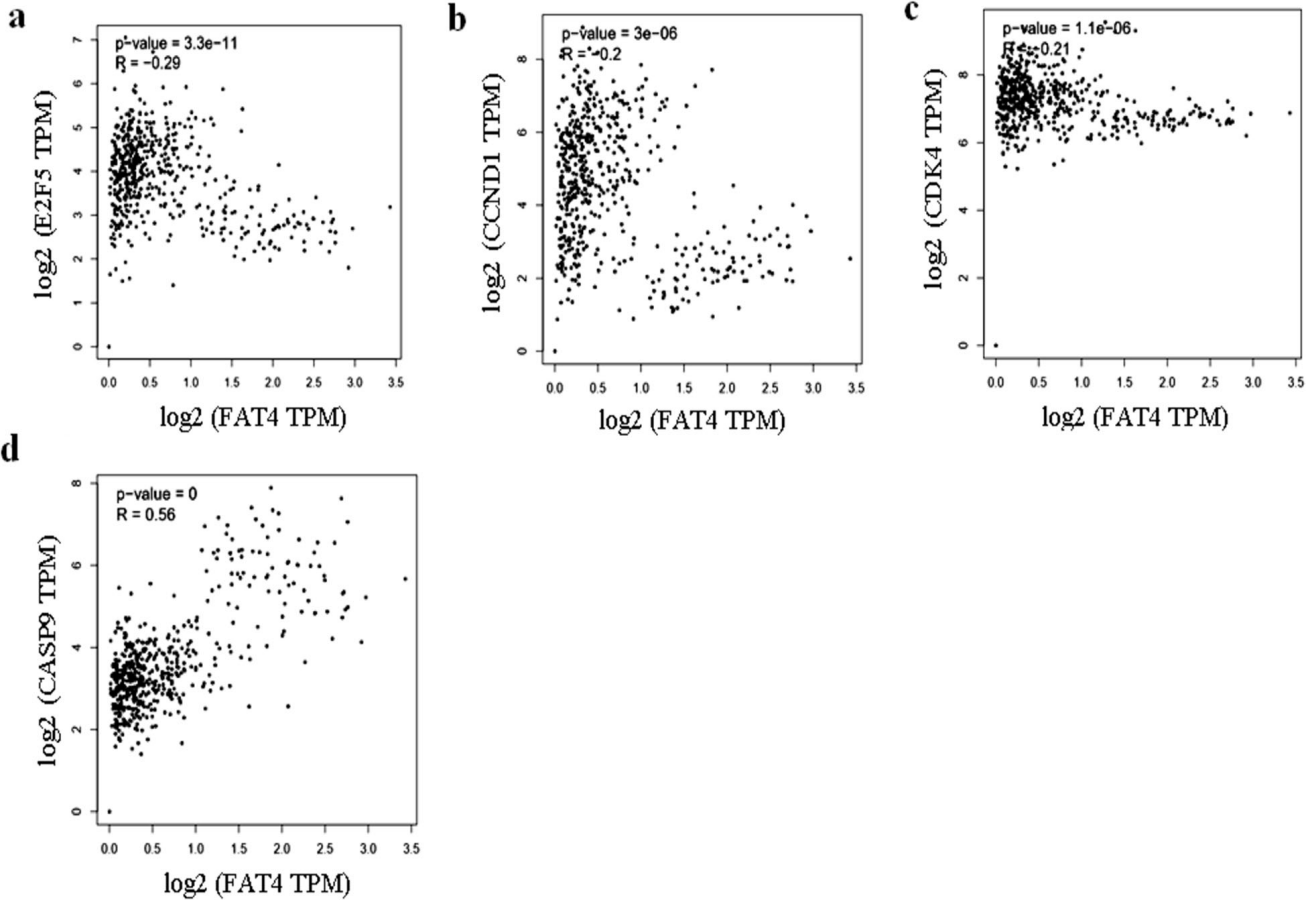
Pearson’s correlation between different gene expressions using samples from GEPIA database. **a**. *FAT4* and *E2F5* is negatively correlated (*p* = 3.3e^− 11^, *r* = − 0.29). **b**. Cyclin D1 expression was negatively correlated with *FAT4* (*p* = 3e^− 6^, *r* = − 0.2). **c**. Pearson’s correlation demonstrating negative correlation between cdk4 and *FAT4* (*p* = 1.1e^− 6^, *r* = − 0.21). d. *FAT4* was positively correlated with caspase 9 (*p* < 0.05, *r* = 0.56)

Consistent with the western blotting results, Pearson’s correlation showed a negative correlation between *FAT4* and *E2F5* (*p* = 3.3 e^− 11^, *r* = − 0.29); (Fig. 5a), Cyclin D1 (*p* = 3e^− 6^, *r* = − 0.2) (Fig. 5b); Cdk4 (*p* = 1.1 e^− 6^, *r* = − 0.21) (Fig. 5c); while a positive correlation was observed between caspase 9 and *FAT4* (*p* < 0.05, *r* = 0.56) (Fig. 5d).

### *FAT4* knockdown promotes E2F5 upregulation

**Fig. 6:**
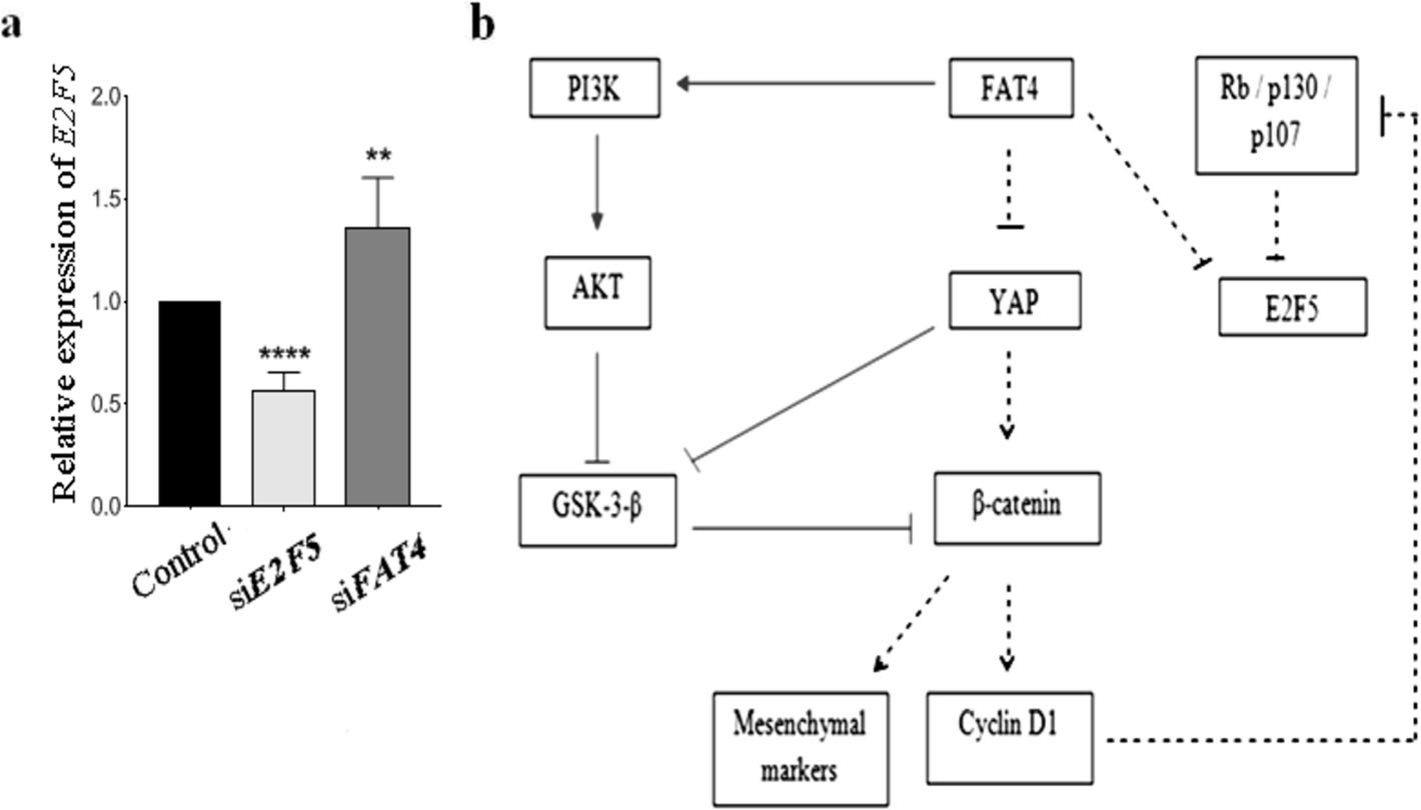
**a**. FAT4 knockdown upregulated the expression of E2F5 suggesting a link between the two genes. **b**. A suggested mechanism of EMT and cell cycle regulation by FAT4 based on Western blot obtained data. Inhibition of FAT4 regulates the expression of EMT markers and promotes cell cycle progression via the suggested pathway. The solid lines indicate the results obtained from the previous study [13], while the dotted lines indicate results of our western blotting experiments

*FAT4*-knockdown cells express a significantly higher amount of *E2F5* suggesting a link between the two genes (Fig. 6a), hence a pathway speculating the mechanism of EOC pathogenesis via *FAT4*, and *E2F5* has been proposed (Fig. 6b).

## Discussion

In this study, we examined the role and associated signaling mechanism of *FAT4* gene in EOC tumorigenesis. *FAT4* expression was weaker in ovarian cancer cell lines, with MCAS and OVSAHO displaying higher expression as compared to A2780 and A2780 cis ovarian cancer cell lines (Fig. 1a and b). Although OVSAHO represents high grade serous ovarian cancer, the expression of *FAT4* was similar to that of MCAS, a low-grade ovarian cancer cell line [25]. The non-correlation between the MCAS cells genotype/phenotype could be an explanation for this contradiction. Indeed, it has recently been shown that, although OVSAHO cells represent high grade serous ovarian cancer, they displayed less potential in invasion, migration, and soft agar colony formation assays [26]. Bioinformatics analysis of Human samples revealed similar results, showing downregulation of *FAT4* expression in Human ovarian cancer samples as compared to normal tissues (Fig. 1c).

In a previous study, immunohistochemical analysis revealed lower *FAT4* expression in gastric [27], colorectal [13], and endometrial cancer [18], when compared to adjacent non-cancerous tissues. Moreover, exome sequencing of *FAT4* in gastric cancer revealed hyper-methylation of the *FAT4* promoter, suggesting that epigenetics is a likely mechanism behind *FAT4* downregulation [28]. Interestingly, *FAT4* downregulation was inversely associated with the tumor grade, and higher repression corresponds to advanced grades [29]. Downregulation of *FAT4* expression was significantly associated with lymph node invasion and poor survival in gastric [12] and endometrial cancer [18]. However, the role of *FAT4* in the pathogenesis of EOC remains still unclear.

To gain further insight into the functional role of *FAT4* on cell viability, colony formation ability and invasiveness, we transfected MCAS and OVSAHO with *FAT4*-siRNA (Fig. 2a, b, c and d) and assessed the proliferation, colony formation, and invasiveness of the cells. We observed that transient reduction of *FAT4* significantly promoted cell growth (Fig. 3a), increased the colony-forming (Fig. 3b and c), and invasive ability (Fig. 3d). These results are in agreement with a previous study in gastric and breast cancer cell lines [8, 12], showing that silencing *FAT4* expression increased invasion and migration of MDA-MB-436 and BT-549 breast cancer cell lines [8], and promoted cell viability, colony-forming ability of BGC-823 and HGC-27 gastric cancer cells [12]. To elucidate the cell proliferation mechanism(s) in *FAT4* siRNA treated gastric cancer cells Ma et al. performed a flow cytometry to reveal an increase in the number of cells in S and G2/M phases, as well as an upregulation of proliferation-associated markers such as cyclin D1, c myc [21] in gastric cancer, and cdk 1 and cdk 2 [18] in endometrial cancer.

Tumor progression involves changes in cell-cell and cell-matrix interactions, allowing the tumor cells to leave the primary tumor and metastasize to distant sites. This complex process seems to be attributed to the loss of epithelial and gain of mesenchymal cadherins, which are considered as a hallmark of EMT [30]. Our results revealed that silencing *FAT4* in EOC cells upregulated the mesenchymal marker (vimentin), and downregulated the epithelial marker (E-cadherin) as compared to control (Figs. 3a-c). These results are in agreement with the reported data obtained in breast and gastric cancer cell lines [8, 12]. In gastric cancer, the expression of MMP-14 and MMP-16 was found to be upregulated, thereby promoting EMT through Wnt/β-catenin pathway [12]. Similarly, in breast cancer, *FAT4* silencing resulted in the upregulation of N-cadherin, MMP-7, and Cyr61 expression and the downregulation of E cadherin, indicating the role of *FAT4* in the EMT process [8]. Furthermore, *FAT4* silencing promoted metastasis in vivo, in gastric cancer xenograft mouse model [12].

The Hippo pathway, regulates cell growth, proliferation, and apoptosis [31]. The downstream effector of this pathway (YAP), is a crucial ovarian cancer oncogene promoting increased cell proliferation, invasion, and anchorage-independent growth [24]. Our Western blotting results revealed higher level of active, unphosphorylated YAP in *FAT4* siRNA treated cells, which promotes proliferation by active transcription of cell growth and anti-apoptotic genes (Fig. 4). Further studies on the nuclear localization of YAP following FAT4 repression are required. The levels of pYAP did not change in our study similar to the results demonstrated by Ito et al., where consistent phosphorylated YAP levels in *FAT4* siRNA treated cells and control MCF-10A breast cancer cells was observed [22], in contrast, pYAP expression was lower in *FAT4* siRNA treated endometrial cancer [18] suggesting the differences in mechanisms ruling these different cancers. In gastric [21] and endometrial cancer [18] cells higher level of nuclear YAP was observed following FAT4 repression.

Wnt/β-catenin pathway plays a crucial role in regulating cell growth and tissue homeostasis, and its activation is a hallmark of several cancers [32]. In this study, the tumor suppressor role of *FAT4* was shown to be mediated by the regulation of β-catenin activity (Fig. 4). Similarly, a previous finding revealed that *FAT4* knocked-down gastric cancer cells [12] displayed higher levels of nuclear β-catenin accumulation as compared to the cytosolic fraction, thereby increasing cell growth over 6 days, while β-catenin repression reduced the growth [12].

GSK-3-β, a component of the Wnt/β-catenin pathway, is known to phosphorylate β-catenin in the cytoplasm, which becomes then targeted for degradation when the pathway is inactive [33]. In a previous study, inhibition of GSK-3-β activity was observed upon phosphorylation at serine-21 in GSK-3-α and serine-9 in GSK-3- β [34–36] while phosphorylation at tyrosine 216 is required for maximal activity [37]. In this study, knocking down *FAT4* downregulated the level of GSK-3-β phosphorylated at serine-9 residue, suggesting further investigation on the levels of GSK-3-β phosphorylated at 216 tyrosine residue, considered as the most active form, are necessary to be able to draw relevant conclusions about its activity following *FAT4* repression. In line with our findings, in colorectal cancer, the downregulation of GSK-3-β upon *FAT4* repression, while upregulation of its phosphorylated form, was observed, which was attributed to the PI3K-AKT pathway in colorectal cancer [13]. Furthermore, YAP overexpression was shown to increase pGSK-3-β and β-catenin, thereby upregulating cyclin D1 expression in glioma [33]. Hence, Wang et al. suggested the ability of YAP to modulate and increase transcriptional activity of β-catenin, demonstrating a link between the Hippo and Wnt pathway [33].

We found that knockdown of *FAT4* resulted in an upregulation of Bcl2 levels. In a previous study, Bcl2 expression was upregulated on YAP overexpression, which could be a possible mechanism of Bcl2 upregulation in our result [38].

Retinoblastoma pathway is a tumor suppressor pathway that plays a key role in regulating the cell cycle, differentiation, and apoptosis [39]. Rb phosphorylation (pRb) by cyclin-dependent kinases (cdks) during G1 phase cause Rb to dissociate from E2F transcription factors resulting in transcription of genes responsible for promoting entry into S phase [40]. In this study, *FAT4* repression increased cyclin D1, and cdk4 expression, promoting progression into the G1/S phase of the cell cycle (Fig. 4a-c), consistent with results obtained in a previous study on gastric cancer cells [21].

In agreement with results obtained on cell lines, bioinformatics analysis also revealed an inverse correlation between *FAT4* and *E2F5* (Fig. 5a). The correlation between *FAT4* and caspase-9, cyclin D1, cdk4 was consistent with that observed in cell lines (Fig. 5b, c, and d). However, there was no significant correlation observed between Rb, and *FAT4*, suggesting an alternative mechanism of regulating cell cycle by *FAT4* through indirect regulation of p107 and p130, members of the retinoblastoma protein family.

Interestingly, *FAT4*-knockdown cells express a significantly higher amount of E2F5 (Fig. 6a), suggesting the tight relationship between these two genes. These data suggest a cross-talk between the two genes (*FAT4*, *E2F5*) and confirm the results obtained by ChIP analysis where *FAT4* was found to be a real target of the transcription factor *E2F5*. E2F5 seems to be suppressed by gene *FAT4*, and its upregulation occurs in cells treated with *FAT4*-siRNA suggesting a link between the two genes.

Based on our western blot results, a plausible role of *FAT4* on the mechanism of EMT, and the cell cycle regulation is proposed (Fig. 6b). Silencing *FAT4* upregulated the expression of unphosphorylated active YAP, which in turn, increases the level of non-phosphorylated active β-catenin. YAP is also known to directly enhance the level of active β-catenin by inhibiting its degradation. Nuclear translocation of β-catenin leads to transcription of cyclin D1, c-myc, and mesenchymal markers such as twist, vimentin, hence promoting cell growth and EMT. Alternatively, *FAT4* can inhibit GSK-3-β and activate β-catenin in part through PI3K-AKT pathway [13].

Furthermore, cyclin D1, a transcriptional target of β-catenin, is known to phosphorylate the retinoblastoma protein family, Rb, p130 and p107 resulting in its inability to associate with *E2F5*, and increase the level of *E2F5* protein. p130 and p107, members of Rb family, are known to block the transcription of *E2F* regulated genes by interacting exclusively with *E2F5* and *E2F4* respectively, which suggests an alternative mechanism of *E2F* activity regulation by retinoblastoma [40, 41]. p107 and p130 are inactivated through phosphorylation by cyclin D1 [42] and cyclin D dependent kinases [43] such as cdk4 [44] at multiple sites, resulting in the transcription of the genes involved in the cell cycle to promote proliferation.

## Conclusions

To the best of our knowledge, this is the first time that *FAT4* tumor suppressor gene is reported in EOC pathogenesis. Our results demonstrated the involvement of *FAT4* in EMT and cell cycle through the regulation of E2F5, YAP, and β-catenin activity. This study provided a better understanding of the anticancer molecular mechanisms of *FAT4,* and we suggest considering *FAT4* gene as a potential target for anticancer therapy by targeting any component involved in its downregulation. These might include epigenetic therapies such as reverting DNA methylation, known to inactivate several tumor suppressor genes, including *FAT4*.

## Supplementary information

**Additional file 1: Supplementary Figure S1.** The original Western blot of fig 1b/2b/2d/4b are displayed.

## Data Availability

All the data generated and/or analyzed during this study are included in this published article (and its supplementary information files), and other datasets will be available from the corresponding author on reasonable request.

## Abbreviations

FAT4: FAT tumor suppressor homolog 4
EMT: Epithelial-to-mesenchymal-transition
EOC: Epithelial Ovarian cancer
ChIP: Chromatin immunoprecipitation
TCGA: The Cancer Genome Atlas
qRT-PCR: Quantitative real time polymerase chain reaction.
